# Elderly Man With Severe Eye Pain After Practicing Eye Drop Technique in Dim Light

**DOI:** 10.1016/j.acepjo.2025.100253

**Published:** 2025-09-20

**Authors:** Hideki Fukuoka, Chie Sotozono

**Affiliations:** Department of Ophthalmology, Kyoto Prefectural University of Medicine, Kyoto, Japan

**Keywords:** medication error, chemical injury, keratoconjunctivitis, clotrimazole, packaging confusion, emergency, antifungal toxicity

## 1 Patient Presentation

**Figure 1:**
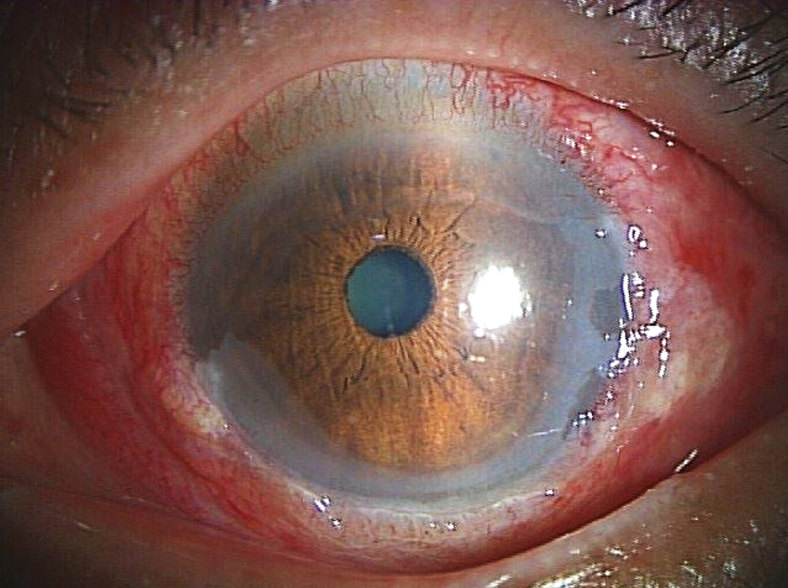
Slit-lamp examination showing severe conjunctival injection with chemosis and mild anterior chamber reaction following accidental clotrimazole instillation.

**Figure 2:**
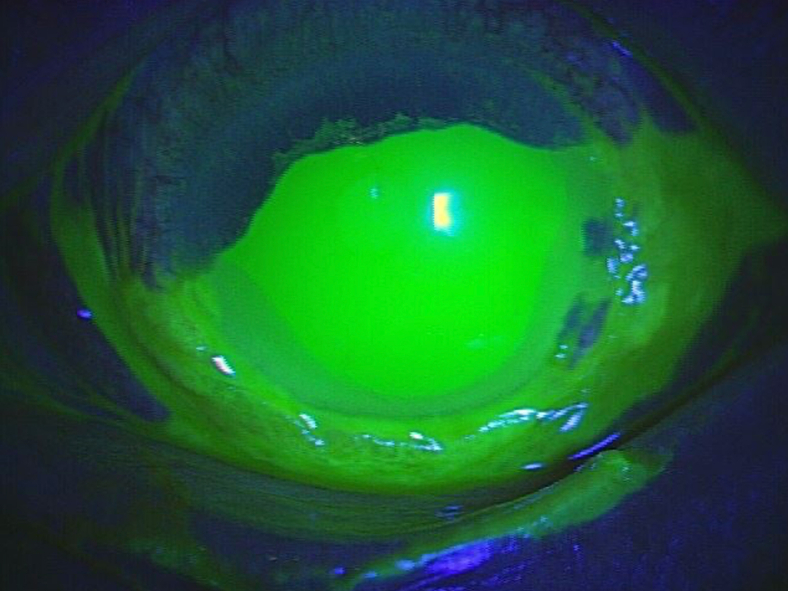
Fluorescein staining demonstrating inferior corneal epithelial defects covering approximately 60% of the corneal surface and conjunctival epithelial erosions.

A 68-year-old male presented to the emergency department with an acute onset of severe eye pain, tearing, and blurred vision after accidentally instilling 1% clotrimazole topical solution into his left eye while practicing preoperative and postoperative eye drop technique in dim lighting conditions prior to scheduled cataract surgery. Visual acuity was 20/80 in the affected eye. Slit-lamp examination revealed severe conjunctival injection with chemosis and mild anterior chamber reaction (Fig 1). Fluorescein staining demonstrated inferior corneal epithelial defects covering approximately 60% of the corneal surface and conjunctival epithelial erosions (Fig 2).

## 2 Diagnosis: Chemical Injury With Keratoconjunctivitis Secondary To Accidental Instillation Of Topical Dermatologic Antifungal Solution

**Figure 3:**
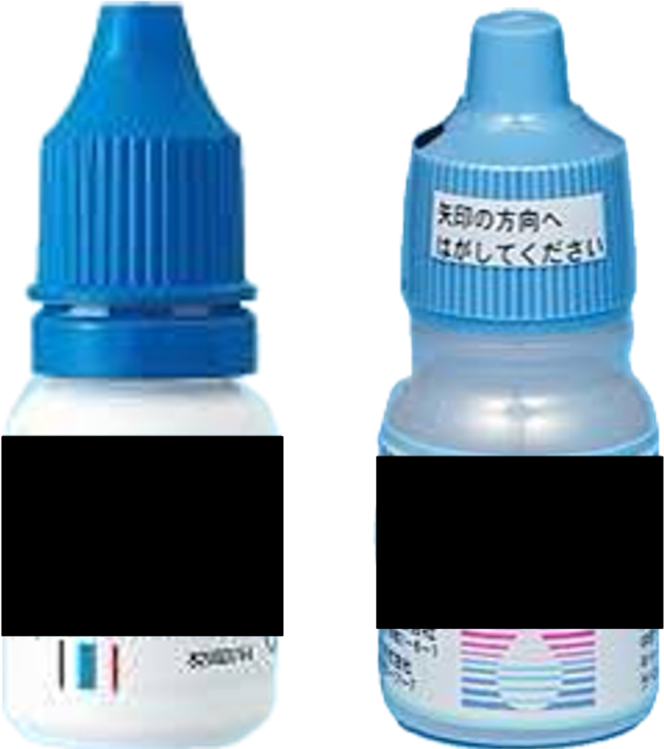
Comparison of packaging between dermatologic antifungal solution (left, brand name masked) and typical ophthalmic eye drop bottle (right), demonstrating striking similarities in plastic dropper bottle shape, blue caps, and overall appearance that contributed to the medication error.

Chemical injuries from inadvertent instillation of topical medications represent an underrecognized cause of ocular emergency presentations. This case highlights critical packaging similarities between dermatologic antifungal solutions and ophthalmic preparations, including identical plastic dropper bottles, similar colored caps, and colorless solutions (Fig 3). These similarities pose significant medication error risks, particularly for elderly patients managing multiple medications in suboptimal lighting conditions.^1^ The Food and Drug Administration has documented similar packaging confusion with topical preparations, noting that similar container shapes and labeling create a substantial risk of confusion.^2^ Treatment with aggressive topical corticosteroids, antibiotic prophylaxis, and artificial tears resulted in complete recovery within 3 months. The excellent prognosis was attributed to immediate recognition, prompt extensive saline irrigation, and timely anti-inflammatory therapy. This case underscores the urgent need for improved pharmaceutical packaging differentiation and enhanced patient education to prevent similar incidents in vulnerable populations preparing for ophthalmic procedures.^3^

## Funding and Support

By *JACEP Open* policy, all authors are required to disclose any and all commercial, financial, and other relationships in any way related to the subject of this article as per ICMJE conflict of interest guidelines (see www.icmje.org). The authors have stated that no such relationships exist.
